# Innovation in health economic modelling of service improvements for longer-term depression: demonstration in a local health community

**DOI:** 10.1186/1472-6963-13-150

**Published:** 2013-04-26

**Authors:** Jonathan Tosh, Ben Kearns, Alan Brennan, Glenys Parry, Thomas Ricketts, David Saxon, Alexis Kilgarriff-Foster, Anna Thake, Eleni Chambers, Rebecca Hutten

**Affiliations:** 1 School of Health and Related Research, University of Sheffield, Regent Court, 30 Regent Street, Sheffield, South Yorkshire, S1 4DA, UK; 2 Sheffield Health and Social Care NHS Foundation Trust, Sheffield, UK

**Keywords:** Cost-effectiveness, Depression, Chronic, Psychotherapy, Mental health services

## Abstract

**Background:**

The purpose of the analysis was to develop a health economic model to estimate the costs and health benefits of alternative National Health Service (NHS) service configurations for people with longer-term depression.

**Method:**

Modelling methods were used to develop a conceptual and health economic model of the current configuration of services in Sheffield, England for people with longer-term depression. Data and assumptions were synthesised to estimate cost per Quality Adjusted Life Years (QALYs).

**Results:**

Three service changes were developed and resulted in increased QALYs at increased cost. Versus current care, the incremental cost-effectiveness ratio (ICER) for a self-referral service was £11,378 per QALY. The ICER was £2,227 per QALY for the dropout reduction service and £223 per QALY for an increase in non-therapy services. These results were robust when compared to current cost-effectiveness thresholds and accounting for uncertainty.

**Conclusions:**

Cost-effective service improvements for longer-term depression have been identified. Also identified were limitations of the current evidence for the long term impact of services.

## Background

The majority of people with depression will experience a full recovery, however for some the outlook is uncertain. This includes people whose depression is a chronic condition (sometimes called ‘treatment resistant’ depression) and those who have recurrent episodes of major depression (also termed ‘relapsing’ depression). Attention was drawn to the importance of this group as early as 1977 [1], and there is a growing body of research specifically focusing on their needs [2]. The awareness that depression for many people is a lifelong condition has yet to make a significant impact on the way mental health services are designed and delivered. Yet efficient services and effective treatments would have significant implications, in terms of health outcomes and service utilisation over a persons’ remaining lifetime [3]. For example, in the UK an Office of National Statistics study in 2000 estimated the total direct costs of depression for the NHS as £370 m, along with significant indirect costs for the economy (£8bn total morbidity costs, £562 m total mortality costs) [4]. It is clear from this study, and other similar cost-of-illness studies [5,6] that depression imposes a significant burden on individuals, carers, the health service and the wider economy. Therefore it is of fundamental importance to ensure that health care resources are efficiently used to maximise health benefits for people with longer-term depression.

The ‘Improving Quality and Effectiveness of Services Therapies and Self-management for longer-term depression’ (IQuESTS) study is a collaborative research project undertaken by mental health service providers, commissioners and health service researchers. It is committed to translating the results of research to routine NHS care, by improving self-management, and demonstrating a step change in the quality and effectiveness of psychological therapies (PT) and services.

There are many potentially cost-effective interventions that may improve the quality and outcomes of services for people with longer-term depression. A number of these are included under the generic term ‘collaborative care’, a systematic approach which supplements primary medical care with mental health interventions from a range of practitioners [7]. Examples with economic evaluations include behavioural activation [8], problem-solving [9], nurse-led self-help [10], family psychoeducation [11], and the UK collaborative and stepped care initiative ‘Improving Access to Psychological Therapies (IAPT) [12]. These evaluations of single interventions or programmes do not meet the requirements of the IQuESTS project for a framework to examine, within a local community, the multiplicity of people’s care pathways through the whole system and to estimate the likely impacts on costs and outcomes of changes to that system.

This analysis is the first of three work packages of the IQuESTS project, where the cost-effectiveness of potential service improvements is estimated. This analysis, together with a qualitative study of service user self-management, has informed the final work package where evidence-based innovations are being tested in routine services.

When considering ways to improve clinical effectiveness and service quality across the whole care pathway in longer-term depression, it is important to be able to estimate which innovations are likely to be cost-effective, as has been demonstrated for specific service configurations, such as collaborative care [7]. A cost-effectiveness analysis is a method of economic evaluation that considers both the additional health benefits and additional costs of a new intervention or service development to determine whether they represent value for money. Health benefits can be measured using Quality Adjusted Life Years (QALYs), a measure that combines length of life with health-related quality of life (HRQL) [13]. HRQL values are scaled, where 1 represents full health and 0 is equivalent to death. An 18 month depressive episode with HRQL of 0.4 would equal 0.6 QALYs. Lifetime QALYs are calculated by aggregating the QALYs for each health state. To compare alternative possibilities, incremental costs and incremental QALYs between comparators are calculated. The ratio of these is called the incremental cost-effectiveness ratio (ICER).

The National Institute for Health and Clinical Excellence (NICE) is an independent organisation in the UK that provides guidance on the use of treatments in the NHS. Although drugs and treatments approved by NICE technology appraisals are mandatory for local services to fund and implement, other NICE guidance (such as public health guidance and clinical guidelines) are not. Local services must take NICE guidance into account but it does not replace the knowledge and skills of individual health professionals who treat patients; it is still up to them to make decisions about a particular patient in consultation with the patient and/or their guardian or carer when appropriate. Resource allocation decisions too are largely discretionary and decision-making on a local level remains vitally important. Cost effectiveness analyses are a key component of that NICE appraisal process of new treatments, and NICE has a threshold range of £20,000 - £30,000 per additional QALY. If a treatment has an ICER above this threshold, then it is unlikely to get a positive recommendation. The NICE threshold may be adopted by local decision makers, in the absence of a local threshold although in practice there appears to be wide variation in local spending decisions [14]. Due to the chronic nature of longer-term depression, an estimate of lifetime costs and benefits is required [15]. Mathematical modelling methods are used to extrapolate short-term clinical trial evidence, as well as incorporating longer term observational data, to estimate lifetime costs and QALYs [16].

Mathematical models have frequently been applied to services of care for patients with depression, including NICE guidance [17] and guidelines [3,18,19]. Some have considered screening for depression [20] and others for recurrence prevention [10,11], However these models are concerned with treatments at one point in a patient’s pathway. Models identified in published systematic reviews were often only short-term, and were not flexible, so do not allow a consideration of the different possibilities at various points in a person’s pathway [21-23]. Tappenden *et al.*[24] have recently developed a ‘Whole Disease Model’ for colorectal cancer which provides a consistent platform through which to evaluate the cost-effectiveness of potential policy changes within the UK colorectal cancer service. This form of modelling moves away from the notion of considering the optimal policy at an isolated point in the broader care pathway, to modelling the pathway itself and the range of decisions therein. In taking this broader perspective, Whole Disease Modelling can be used to address a much wider range of questions considering the configuration of health services with the intention of improving overall health outcomes across the system.

Tappenden *et al.*[24] have developed a methodological framework for Whole Disease Modelling, and this analysis draws upon this framework, to enable the evaluation of multiple changes to the care pathway for depression treatment in the NHS.

## Methods

The aim of this study was to demonstrate the use of whole system cost effectiveness modelling within a health community. Sheffield, a UK city in South Yorkshire, has an NHS Foundation Trust providing mental health services and 109 primary health care centres serving a population of 555,500. Its Foundation Trust status means it has managerial and financial independence from the Department of Health and local NHS health authorities with regards to how services are provided. Ethical approval was granted by the National Research Ethics Service (NRES) for the study (REC 10/H1310/51). Only anonymised routine NHS data were used in the analysis and as such participant consent was not required.

### Conceptual model

The first stage of the process was to develop two conceptual models, through interviews with local mental health service experts (managers, service users, clinicians and academics), as well as from the relevant research literature.

**Figure 1:**
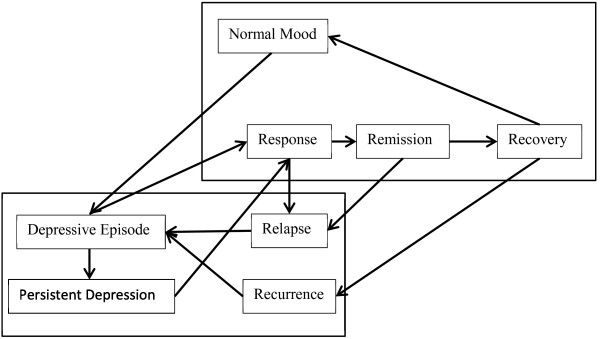
Disease level conceptual model.

First, a disease-level conceptual model was developed (Figure 1). Longer-term depression is characterised by a person relapsing from remission or recovery into a depressive episode (recurrent depression), or by a treatment failing to see an improvement in a person’s condition (treatment-resistant/persistent depression). Whether a person is in response, remission or recovery is dependent on how long their normal mood has lasted since a depressive episode.

Secondly, a service-level conceptual model was developed to describe the delivery of psychological therapy services for people with longer-term depression in Sheffield, England. At this stage, and at intervals throughout the study, an expert workshop was convened consisting of mental health service commissioners, primary and secondary care clinical staff, service managers, academic specialists and service users. The function of the expert workshop was to advise on the conceptual model and to generate consensus on priorities for evidence-based innovations.

In general, Sheffield has adopted the stepped care model (SCM) as recommended in the 2009 NICE Clinical Guideline for Depression [3]. The SCM is a structured pathway of care for people with depression, directing people to the most appropriate and least costly interventions. The SCM spans primary, secondary and tertiary care services in Sheffield, with access points across medical, third sector and public sector services. Improving Access to Psychological Therapies (IAPT) services with low and high intensity PT are provided in primary care, along with medical therapies provided by general practitioners before IAPT is initiated. Community Mental Health Teams (CMHTs) in secondary care provide more complex and comprehensive medical treatment and PT for people with longer-term depression. People can also step up to tertiary care and the Specialist Psychotherapy Services (SPS) which includes a team focussed on treatment of severe and complex depression.

### Mathematical model

The conceptual model was agreed by the expert workshop as the basis for the fundamental assumptions of the mathematical models’ structure. The mathematical model reflects the relapsing/remitting characteristic of longer-term depression by simulating these two possible health states as evidenced in the disease level conceptual model. The mathematic model assigns probabilities to a patient moving from one point to another, simulating patient pathways through the service system. These probabilities are calculated using evidence from a range of sources, which allows the model to estimate the costs and health benefits of the current care pathway.

The estimates of costs and health benefits provide a baseline estimate of efficiency within the service. The model was then used to estimate the impact of changes to the service i.e. does a change to the service affect health benefits and costs? The model was therefore used to estimate if changes to the service would be cost-saving, or cost-effective.

**Figure 2:**
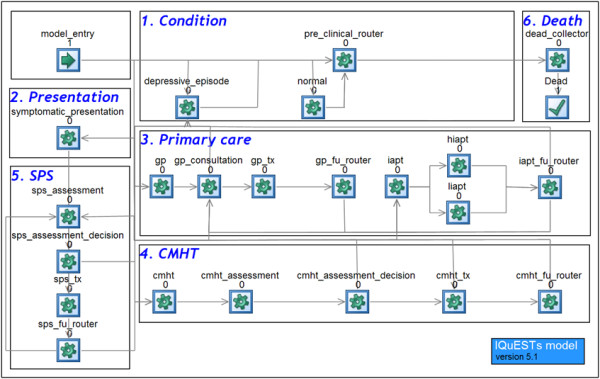
Simulation model.

A discrete event simulation model was developed in Simul8©. This methodology estimates the costs and QALYs for unique simulated people, and after running 1,000 simulated people the average cost and QALY per person are calculated. This approach is different from a cohort/Markov model methodology, because individual patients with unique characteristics are simulated, rather than an average cohort. This overcomes the limitations of Markov models and allows a greater degree of complexity to be incorporated [25]. Both costs and QALYs are discounted at 3.5% each year, in line with current guidance to account for time preferences [15]. The simulation of the patient pathway is shown in Figure 2. The simulation model has work centres for each event. A work centre contains logic to determine movements to future work centres (i.e. a movement from a GP consultation to a GP treatment decision). Work centres also update the costs and health benefits for a patient as they are simulated. In Box 1 (Condition) of the simulation model, patients are simulated to be in a normal state or a depressive episode. If they have a depressive episode, then they can present to NHS services via Box 2. The patient can then step through primary care treatment, IAPT therapies (Box 3), CMHTs (Box 4) and tertiary SPS (Box 5). Each treatment step begins with an initial assessment to determine if treatment is appropriate. If so, the patient is treatment, and their subsequent route through the model is then determined based on their response to treatment. When a depressive episode ends, either naturally or due to successful treatment, they return to the normal state work centre in Box 1. The simulated patients are modelled until they die and proceed to Box 6.

### Comparators

The IQuESTS expert workshop generated consensus on evidence-based innovations for service improvement. From many suggested topics, twelve were selected for discussion at a workshop and eight were included in a Delphi exercise. From the workshop and Delphi, three were selected for evaluation in the model by the research team via a discussion based on their ease of modelling. One of the workshops was also used to validate the conceptual and mathematical models.

#### Self-referral back to therapy after discharge

In current services, after discharge from the psychological therapy service, a new episode of depression would require re-referral back to the team via the GP, necessitating a further wait. In this service change, self-referral can occur if the next depressive episode happens within 6 months of a response to PT. It costs an extra session and the person is fast-tracked back into a course of PT. It is assumed that the details are provided to the person on a contact card on discharge from the service. At the workshop, experts commented that this is sometimes informally provided. The main anticipated benefit is the reduction in time to re-enter PT for people with relapsing depression.

#### Better management and prevention of drop-out from psychological therapy

Almost 50% of people will drop out of PT [26,27]; however interventions to address this significant problem have not been widely developed. There is some evidence to suggest that psychotherapy dropouts might be minimised if differences between therapists’ and patients’ perspectives on the therapeutic enterprise are acknowledged and recognised as legitimate targets for intervention. This supports an additional therapist session specifically to address these issues and to develop collaborative strategies with the aim to reduce dropout [28-30]. The model simulates this service change by a 20% reduction in the probability of drop-out in primary, secondary and tertiary PT. This costs one therapist session, for the development of management and coping strategies. The modelled assumptions of costs and effectiveness were considered and agreed by the expert workshop.

#### Widening access to non-therapy services

Service users reported benefit from accessing a range of non-therapy services that fall outside traditional PT and medical services, including social groups, exercise, meditation, complementary, physical and creative therapies. Widening access to these through NHS signposting may enhance self-management and see more suitable and beneficial care being provided. The model simulates this service change via a 10% reduction in drop-out, and a 10% increase in response to treatment. The service costs one therapist session. It is assumed that the NHS is only signposting a patient to these services, and not funding a course of non-therapy support. The modelled assumptions of costs and effectiveness were considered and agreed by the expert workshop.

### Model population

The population is defined as people using longer-term depression services. This has been more explicitly defined as people with a diagnosis of recurrent or chronic depression, or more than two separate depressive episodes (ICD codes F32, F33, F34, F38.1 and F41.2). Patients identified could have comorbidities; however depression was the primary diagnosis for the routine NHS data. Each patient is assigned a number of characteristics which determine their route through the model. These characteristics included their current disease status (depressive episode or normal state), age, health related quality of life, and previous history in the model (number of previous depressive episodes, and response/non-response to previous treatments).

The model estimates a ‘natural history’ for each simulated person. The time spent either depressed or well is modelled without the impact of any health services or interventions. This represents the profile for a person who does not engage with services, or the subsequent profile for someone who drops-out of the system. Effective treatments impact on the natural history of the condition by reducing the severity of the episode, shortening the episode, or increasing the time until a relapse. A treatment can be effective in multiple ways, and increasing the time until relapse has long-term health benefits. A mortality impact of longer-term depression has not been modelled, with life expectancies taken from published life tables [31].

### Data

All data parameters and sources are provided in Table 1. Evidence to inform the model was obtained from a range of sources. Resource and time constraints prevented a full systematic review of the literature, and therefore a non-systematic rapid review surrounding the diagnosis, treatment and follow-up of longer-term depression was undertaken. We also searched NICE guidance regarding depression, and the NICE Clinical Guideline for depression was a major source of systematically reviewed evidence [3]. NHS Reference Costs were used to identify costs of services, and routinely collected data from the local mental health trust were analysed for this model. Only NHS costs were incorporated, with the analysis therefore taking a funder perspective. Where data were not available, clinical expert opinions were used to provide informed assumptions.

**Table 1:** Model parameters

**Parameter**	**Value**	**Evidence source and type**
**Epidemiological**		
Length of untreated episode of recurrent depression	3rd episode: 0.55 years.	[32] 10 year observational study
	4th episode: 0.60 years	
	5th episode +: 0.43 years	
Probability of no future recurrence of depression	2 prior episodes: 0.3.	[33]
	3+ prior episodes: 0.1	
Time to relapse	0.93 years	[34] 15 year observational study
**Clinical presentation**		
Probability of presenting to services during depressive episode	0.40	[35] Consensus document
Probability of no relapse after cognitive behavioural therapy (CBT) response	0.75	[36] Randomised controlled trial
**Primary care**		
Probability of medical therapy (even if stepping up)	Assumed 100%	Clinical expert assumption
Probability of response after 8 weeks of mirtazapine anti-depressant therapy	0.63	[3,37] NICE Clinical Guideline
Probability of IAPT referral	Assumed 100%	Clinical expert assumption
**IAPT**		
Probability of low intensity IAPT	0.68	SHSC data
Probability of high intensity IAPT	0.32	
Probability of effective low intensity IAPT (> 10 point improvement in PHQ9)	0.24	
Probability of effective high intensity IAPT (> 10 point improvement in PHQ9)	0.54	
Probability of completing low intensity IAPT	0.35	
Probability of completing high intensity IAPT	0.35	
Number of low intensity IAPT sessions	3 + 1 assessment	
Number of high intensity IAPT sessions	7 + 1 assessment	
Length of course of low intensity IAPT	0.42 years	
Length of course of high intensity IAPT	0.71 years	
**Community Mental Health Teams**		
Time from referral to assessment	0.08 years (4 weeks)	Clinical expert opinion
Probability of accepted at assessment	0.8	
Treatment effectiveness	CMHT treatment effectiveness assumed equal to IAPT	
**Specialist Psychotherapy Services**		
Probability of being accepted at SPS assessment	0.71	SHSC data
Treatment effectiveness	SPS treatment effectiveness assumed equal to IAPT	Clinical expert opinion
**Costs**		
GP	£38	2009 NHS Unit Costs
IAPT Therapist (per session)	£88	2010 NHS Reference Cost
CMHT Assessment	£212	
CMHT Therapist (per session)	£135	
SPS Assessment	£139	
SPS Therapist (per session)	£139	
Mirtazapine treatment. 30 mg daily, 8 week course which if effective is maintained	£4.08 per course	BNF 61
**Health Utilities**		
Untreated severe depression	0.30	[3,38] NICE Clinical Guideline/Cohort analysis
In health service, severe depression	0.58	
Minimal depression/normal health	0.85	

### Analysis

The model simulates 1,000 people, to provide an estimate of the mean cost and QALYs per person. Probabilistic Sensitivity Analysis (PSA) was undertaken to quantify the uncertainty in the parameter data, and this was performed by running 500 additional simulations drawing from assigned distributions for each parameter input. In particular, Weibull distributions were applied for the epidemiological parameters, beta distributions for probabilities and utilities, and uniform distributions for the NHS reference costs. Analyses were performed to ensure that these were an appropriate number of simulations. Validation of the model was performed by testing the model code and checking results and this was performed by a second analyst. Error checking was undertaken, and the face validity of the model was constantly tested by the project team and steering group. Scenario analyses were performed, by changing model assumptions to test the sensitivity of the cost-effectiveness results. A factorial design analysis was undertaken to evaluate the impact of undertaking multiple service changes at the same time.

## Results

### Service impact

Table 2 provides more detailed results regarding the impact of the service changes on the longer-term depression health service. The table also shows that on average, a person experiences nine depressive episodes. The results for visits to primary, secondary and tertiary are for complete courses of care, and so on average people are much more likely to receive care in primary services (2.9 courses of treatment) rather than secondary (0.1 courses) or tertiary (0.05 courses). The table shows that there is a small reduction in the number of depressive episodes a person will experience on average.

**Table 2:** Service impact results

**Model**	**Episodes**	**System results**			**PT courses**	**Costs (undiscounted), £**			
		**Primary visits**	**Secondary visits**	**Tertiary visits**		**Primary**	**Secondary**	**Tertiary**	**Total**
Baseline	9.283	2.994	0.148	0.048	1.398	£666	£171	£50	£886
Self-referral	9.196	2.486	**0.160**	**0.062**	**1.493**	**£759**	**£208**	**£78**	**£1,046**
Reduce drop-out	8.754	2.788	**0.192**	**0.077**	**1.423**	**£760**	**£244**	**£95**	**£1,099**
Non-therapy	8.823	2.793	0.118	0.026	1.288	**£716**	£143	£31	**£891**

Values in **bold** show an increase compared to baseline.

The self-referral service change, as expected, predicts a reduction in primary care visits, with people instead being fast tracked back to secondary and tertiary services. The number of PT courses being completed increases. This is due to people bypassing antidepressant treatment offered in primary care, and moving straight back to PT. The total effect is an increase in the costs attributed to each level of the service. The increase in primary care costs is mostly driven by the increase in high intensity IAPT. This service change bypasses GP treatment and antidepressant therapies, which are modelled as effective in this patient population, and have lower drop-out rates than PTs.

The reduce drop-out service change has the expected effect of increasing visits across secondary and tertiary services. It also results in a greater number of psychological therapy courses being completed. The total effect is an increase in the cost of providing services across all levels.

The increase in access to non-therapy services sees negligible change in service use, which results in only a slight increase in total costs when compared to the basecase. This may be due to the way in which non-therapy services have been modelled, and this will be discussed further in the Discussion section. This is the only service change which results in a cost saving for any individual part of the pathway (secondary and tertiary care), due to patients having less time with untreated depression, and so they do not step up in the system.

### Cost effectiveness

Table 3 provides estimates of the costs and QALYs generated by the baseline configuration, and the three service changes. The results presented are from the probabilistic sensitivity analysis (PSA), and are discounted, and the incremental analysis for each service change is compared to the basecase model.

**Table 3:** Cost-effectiveness results

**Model/service change**	**QALYs**	**Cost (£)**	**Cost-effectiveness (compared to current care)**		
			**Incremental QALYs**	**Incremental costs**	**ICER**
Current care	15.023	£699.56	-	-	-
Self-referral	15.034	£827.02	0.011	£127.46	£11,378
Reduce drop-out	15.110	£893.87	0.087	£194.30	£2,227
Non-therapy	15.096	£716.03	0.074	£16.47	£223

All three service changes result in additional QALYs, when compared to current care. These additional QALYs come at an increased cost for all three changes. The self-referral change is both more effective (+0.011 QALYs) and more costly (+£127), which results in an ICER of £11,378 per additional QALY, when compared to current care. The reduce drop-out change generates the greatest additional QALYs (+0.087), but also at an additional cost (+£194), which results in an ICER of £2,227 per additional QALY, when compared to current care. The non-therapy change is more effective (+0.074) and more costly (+£16), which results in an ICER of £223 per additional QALY, when compared to current care.

**Figure 3:**
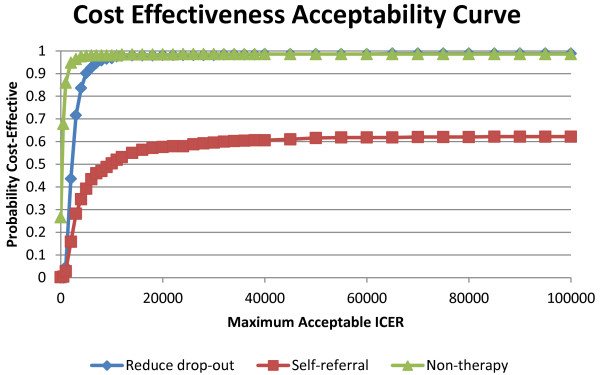
Cost effectiveness acceptability curve.

Figure 3 presents the PSA results in the form of a cost effectiveness acceptability curve (CEAC), which indicates the probability that a service change is cost-effective for a range of ICER thresholds when compared to current practice. The CEAC shows that both the reducing drop-out and non-therapy service changes are likely to be cost-effective at a £20,000 per additional QALY threshold compared to current practice (probability > 0.98). At this threshold level, the self-referral change has a probability of being cost-effective of 0.57.

The CEACs highlight that there is uncertainty in the model parameters, which results in uncertainty as to whether a service change will be cost-effective. The 500 PSA simulations provided estimates of negative incremental QALYs for all three service changes. Only a small number of simulations for the non-therapy service change resulted in negative incremental costs (i.e. saved money). This suggests that all three options are likely to require additional NHS resources for implementation.

### Scenario analysis

Scenario analysis was undertaken on the self-referral service change, by varying the time for which self-referral is an option. The basecase time limit for self-referral is 6 months, and this was changed to 3, 9 and 12 month limits. Extending the time limit to 12 months marginally worsened the incremental cost-effectiveness ratio of the self-referral service change (ICER £3,968 per QALY compared to current care).

For the reduce drop-out service change, scenario analyses were undertaken by varying the percentage reduction in drop-out assumed by the new service. The results were in general insensitive to varying the reduction in drop-out between 5% (£5,835 per QALY) and 25% (£2,175 per QALY). The results for the non-therapy service change were also relatively insensitive to changes in the drop-out rate and treatment efficacy.

### Factorial analysis

A factorial design was undertaken with all possible combinations of the three service changes being ‘on’ or ‘off’. This therefore examines if there are any interactions between the services and if a combination of service changes offer greater value for money.

Table 4 presents a fully incremental analysis of the cost-effectiveness results from the factorial design analysis. As expected, the basecase configuration is least effective (14.912 QALYs). Factor combinations 3, 6 and 5 are dominated by (are less effective and more costly than) factor combination 7. The ICER for factor 7 compared to factor 4 (the next less effective and non-dominated comparator) is £5,533 per additional QALY.

**Table 4:** Factorial design results

**Design**	**Self-referral**	**Drop-out reduction**	**Widening non-therapy**	**QALYs**	**Costs**	**Incremental analysis**	
						**ICER**	**Comparator**^**a**^
1	-	-	-	14.912	£832	-	
2	-	+	-	14.935	£1,512	Dominated	Compared to 4
4	+	-	+	14.956	£891	£1,335	Compared to 1
3	+	-	-	14.964	£1,166	Dominated	Compared to 7
6	-	-	+	14.986	£1,568	Dominated	Compared to 7
5	+	+	-	15.002	£2,065	Dominated	Compared to 7
7	-	+	+	15.019	£1,150	£5,533	Compared to 4
8	+	+	+	15.042	£1,993	£24,586	Compared to 7

Results ordered from smallest to largest QALYs - = factor not active + = factor active.

^a^ Comparison against the next less-effective non-dominated option.

The factor combination 2 is dominated by factor combination 4. Compared to the basecase, factor combination 4 has an ICER of £1,335 per additional QALY. Factor combination 8 has all three service changes active, and is the most effective factor combination. Compared to factor combination 7 it has an ICER of £24,586 per additional QALY. It is important to note that factor combination 8 is not the most costly option, which instead is factor combination 5. This is interesting because each individual service change has a cost impact for the NHS. Having a total cost lower than an option with only two service changes suggests that important interactions in the service are occurring when multiple changes are made.

## Discussion

The results suggest that all three proposed service changes would provide health benefits, accounted for by increasing the use and completion of psychotherapy services. As well as the immediate benefit of reducing the length of a depressive episode, the service changes have the long-term benefit of reducing the probability of future episodes of depression. These additional benefits however come at an increased cost for the NHS, due to increased utilisation of services and psychological therapies. The sensitivity analyses (probabilistic, and scenario analysis) undertaken suggest that these results are robust to the model assumptions. The factorial design provides evidence for policy makers when considering multiple potential changes, but also highlights that unexpected interactions may occur and should be carefully considered.

Due to the local nature of the research and decision-maker environment, there is not a defined cost-effectiveness threshold based on local spending and outcomes. This means that interpretation of the cost-effectiveness results cannot be undertaken by this research team, but will need consideration by NHS commissioners and providers. Whilst some national guidance from NICE is mandatory, NICE has only considered a sub-set of possible interventions and treatments and so local policy-making requires evidence to inform it. The use of UK specific data and taking a UK perspective does limit the results of this analysis to a UK context; however we anticipate that the methodologies and concepts will be transferable to other countries.

The results of the analysis highlight the flexibility and appropriateness of the Whole Disease Modelling framework when looking to evaluate service changes which will have a wide and long-term impact. Each of the three service changes was evaluated in terms of their impact across the NHS, and for a patient’s lifetime.

The quantified model also provides useful service performance metrics, such as flows of people through different parts of the services, and changes can be evaluated in terms of their impact on specific resources. This is important because the local NHS is currently going through a period of change, and in general budgets are being restricted.

One important result is that the model suggests only low numbers of people with longer-term depression ‘step up’ to secondary (CMHT) and tertiary (SPS) services. This is because only a small proportion will present, and also because by the time they reach these higher steps, the depressive episode may have passed without a healthcare intervention. In particular, the self-referral service change highlights that if people can quickly receive appropriate therapy then there are likely to be significant health benefits both immediately, and in the longer term.

As with any health economics modelling, there are limitations of the analysis which should be noted. First, the analysis of the local NHS dataset has only been to identify broad flows of patients in the service, and covariates such as ethnicity and severity of depression have not been incorporated into the analysis. Secondly, health economic modelling allows evidence from different sources to be synthesised so that an estimate of the long term costs and benefits of new interventions can be found. As such, the model is limited by the quality of evidence that is available, and by the assumptions used when evidence is missing. The basecase model results suggest that patients experience on average nine depressive episodes in their lifetime. This is derived from longitudinal studies which estimate a time to relapse after a particular number of episodes, however evidence regarding relapse beyond five episodes was not identified, and so the model simulates a potentially inaccurate number of episodes. Experts suggest that this number may be too high, however it should be noted that there is still a low probability of patients presenting, and so these episodes may go undetected.

There is a significant evidence gap regarding the effectiveness of therapies at the higher steps (CMHTs and SPS). For this attempt at modelling the service, the effectiveness of SPS psychological therapy has been assumed no better than CMHT input. Because the IQuESTS project includes a specialist depression research clinic to pilot these service improvements, many of these assumptions can be tested and this will benefit the model in terms of collecting long term data regarding patients’ experiences with depression, and the impact of specific service improvements. This data will be used in an updated version of the model once the pilot study has been completed.

Mortality and serious adverse events have not been incorporated into the model. People who suffer a severe depressive episode have a significantly increased suicide risk, as well as increased risk of self-harm resulting in the need for hospitalisation [3]. Also many people with depression will never access health services. Better access to services for these people will enable effective care and services to be provided, leading to improved health outcomes.

Because the service changes that have been evaluated are novel and un-evidenced, assumptions have been required to incorporate these changes into the model. Relatively simplistic assumptions have been required, such as the cost implications of the changes, and how they may impact on the flows of people through the system. The next step of the IQuESTS project is to explore fully how these service changes will be implemented in a pilot study. These results will inform the further development of the service changes, but may also allow an opportunity for refining the assumptions in the model and improving the accuracy of the results.

The Whole Disease Model framework provides a useful set of stages for a research project of this type. Because a change to a system can impact on other services, it is important to have a wide boundary and ensure that all possible costs and benefits are incorporated into the analysis. This aids decision-makers and commissioners, who are often challenged when provided with economic analyses which have a very specific boundary (i.e. a pairwise evaluation of treatments which does not capture the full care pathway). Also, the Whole Disease Modelling framework allows multiple evaluations of service changes to be undertaken. At this stage, three different service changes have been evaluated, however more can be added relatively easily, which means the Whole Disease Modelling framework allows efficient use of one (potentially more complex) model, rather than requiring several models.

## Conclusions

This study has used the novel Whole Disease Modelling framework to allow a single mathematical model to evaluate several changes to the Sheffield mental health NHS service for people with longer-term depression. The output of this research suggests that reducing drop out, widening access to non-therapy services, and allowing self-referral may have health benefits for people with longer-term depression. However these additional health benefits may be at an increased cost for the NHS. With research currently being undertaken to examine these changes in clinical practice, we have used modelling methods to translate evidence into service change and health improvements for patients. The research has also shown that the Whole Disease Modelling framework is a robust and efficient method for modelling multiple service changes using a coherent and validated mathematical model.

## Competing interests

The authors have no conflicting interests to declare.

## Authors’ contributions

JT, AB, GP and TR designed the original research project. JT, BK and AB undertook the health economic modelling, with DS, AKF and AT assisting with data analysis and model parametrisation. EC facilitated service-user involvement and RH provided project management. JT wrote the manuscript, and all authors read and approved the final manuscript.

## Pre-publication history

The pre-publication history for this paper can be accessed here:

http://www.biomedcentral.com/1472-6963/13/150/prepub
